# Complete Genome Sequence of a Microcystin-Degrading Bacterium, Sphingosinicella microcystinivorans Strain B-9

**DOI:** 10.1128/MRA.00898-18

**Published:** 2018-09-06

**Authors:** Haiyan Jin, Tomoyasu Nishizawa, Yong Guo, Akito Nishizawa, Ho-Dong Park, Hajime Kato, Kiyomi Tsuji, Ken-ichi Harada

**Affiliations:** a Graduate School of Environmental and Human Science, Meijo University, Tenpaku, Nagoya, Japan; b College of Agriculture, Ibaraki University, Ami, Ibaraki, Japan; c GeneBay, Inc., Yokohama, Kanagawa, Japan; d Department of Environmental Sciences, Faculty of Science, Shinshu University, Matsumoto, Japan; e Tohoku Medical and Pharmaceutical University, Sendai, Miyagi, Japan; f Kanagawa Prefectural Institute of Public Health, Chigasaki, Kanagawa, Japan; g Faculty of Pharmacy, Meijo University, Tenpaku, Nagoya, Japan; Loyola University Chicago

## Abstract

Sphingosinicella microcystinivorans strain B-9 has the ability to degrade cyanobacterial hepatotoxic cyclic peptides, microcystins, and nodularins. This is the first report of the complete genome sequence of the microcystin-degrading bacterium.

## ANNOUNCEMENT

The microcystin-degrading Sphingosinicella microcystinivorans strain B-9, which belongs to the family Sphingomonadaceae, was isolated from Lake Tsukui, Japan (1), and the 16S rRNA sequence of strain B-9 was phylogenetically similar to the S. microcystinivorans strain Y2^T^ (2). The strain B-9 completely detoxifies microcystins (3), and the *mlrA*, *mlrB*, *mlrC*, and *mlrD* genes are involved in the degradation of microcystin (4, 5). The *mlrA* and *mlrD* genes are located between *mlrC* and *mlrB*, in which *mlrA* and *mlrC* encode a metallopeptidase, *mlrB* encodes a serine protease, and *mlrD* encodes a putative transporter protein (4). These enzymes are also responsible for the degradation of many physiologically active peptides (6–8) in addition to the hepatotoxic nodularin and nontoxic aeruginopeptin (9, 10). To identify the construction of the microcystin-degrading gene, Okano et al. (11) reported the whole-genome sequence of the microcystin-degrading bacterium Sphingopyxis sp. strain C-1, isolated from a lake in China. We now report the complete genome sequence of the microcystin-degrading S. microcystinivorans strain B-9.

The genomic DNA was isolated from the S. microcystinivorans strain B-9 grown on a LB agar plate using a previously described procedure (12). A 20-kb genomic library was generated, and PacBio RS II sequencing was carried out. A total of 100,841 reads with an average length of 11,613 bp was obtained for a total of 1,117,081,611 bases of sequence. The reads were assembled using Hierarchical Genome Assembly Process 2 (HGAP2) to produce one polished circular contig with a 290× depth of coverage. The genome sequences were annotated using the Dodd-Frank Act Stress Test (DFAST) (13, 14), followed by manual annotation of the predicted hypothetical proteins with the NCBI nonredundant protein database using the BLASTP program (15). The complete genome sequence of strain B-9 consists of a circular 4,036,662-bp chromosome and a GC content of 63.9%, with 3,810 coding sequences, 3 rRNA genes, and 49 tRNA loci.

The microcystin-degrading gene cluster was identified, and the genetic arrangement was *mlrCADBEF*. According to the BLASTP analysis, the amino acid sequences of the genes *mlrA* and *mlrD* share a 100% identity with those of Rhizobium sp. TH, which is one of the root nodule bacteria (16), and 89% and 90% identity with those of strain C-1, respectively. Additionally, those of *mlrB* showed an 89% identity with those of strain C-1 and Sphingomonas sp. strain USTB-05, which belong to the microcystin-degrading bacteria (17). The *mlrE* and *mlrF* genes in strain B-9 were similar to the dipeptidase and D-aminoacylase of strain C-1 (11) and shared 90% and 91% identity to those of strain C-1, respectively. In our previous study, we elucidated that a putative protease MlrE, which hydrolyzes a peptide into amino acids, played a role in the final step of the microcystin-degrading pathway (18). The activity of MlrE was not inhibited by the chelating agent EDTA, suggesting that the responsible enzyme was not MlrC in the final step (4). The strain B-9 genome sequence analysis will contribute to further understanding a system of hydrolytic and transporter enzymes involved in the degradation pathway of microcystin.

### Data availability.

The genome sequence of Sphingosinicella microcystinivorans strain B-9 and the raw read data have been registered in the DDBJ/EMBL/GenBank databases under the accession number AP018711 and in the DDBJ sequence read archive (DRA) under accession number DRA007096.
